# Supplementary material for: Evidence of monkeypox virus clade IIb lineage A.2.2 in the Republic of the Congo and co-circulation of clade Ia, Ib and clade IIb
Source: Nat Med. 2026 Apr 3;32(5):1666–70. doi: 10.1038/s41591-026-04256-2 (PMC13190315; doi:10.1038/s41591-026-04256-2)
Supplement: Supplementary file 1 — Supplementary Tables 1 and 2. [file 41591_2026_4256_MOESM1_ESM.pdf]

nature medicine

Supplementary information https://doi.org/10.1038/s41591-026-04256-2

Evidence of monkeypox virus clade IIb lineage A.2.2 in the Republic of the Congo and co-circulation of clade Ia, Ib and clade IIb

In the format provided by the authors and unedited

Supplementary material

Supplementary Table 1. Model selection for Mpox genomes

Model	PS-logML	SS-logML	PS-Rank	SS-Rank	PS-BF	SS-BF
Strict, Exponential	-253420.94	-253420.67	1	1	0.00	0.00
Strict, Constant	-253423.46	-253423.2	2	2	2.53	2.53
Strict, SkyGrid	-253424.28	-253424.02	3	2	3.34	3.35

#PS=Path sampling, SS= Stepping-stone sampling, BF=Bayes Factor

Supplementary Table 2. MPXV full genomes used in this study

Sequence name	GISAID name	GISIAD accession ID	Clade
hMpxV/RoC/Sangha/2018/01	hMpxV/Congo/LNSP-01/2018	EPI_ISL_19350687	Ia
hMpxV/RoC/Sangha/2018/02	hMpxV/Congo/LNSP-02/2018	EPI_ISL_19350688	Ia
hMpxV/RoC/Sangha/2022/03	hMpxV/Congo/LNSP-03/2022	EPI_ISL_19350689	Ia
hMpxV/ROC/Cuvette-Centralle/2024/01	hMpxV/Congo/LNSP-018/2024	EPI_ISL_19345030	Ia
hMpxV/ROC/Cuvette-Centralle/2024/02	hMpxV/Congo/LNSP-07/2024	EPI_ISL_19345019	Ia
hMpxV/ROC/Cuvette-Centralle/2024/03	hMpxV/Congo/LNSP-05/2024	EPI_ISL_19345017	Ia
hMpxV/ROC/Cuvette-Centralle/2024/04	hMpxV/Congo/LNSP-016/2024	EPI_ISL_19345028	Ia
hMpxV/ROC/Cuvette-Centralle/2024/05	hMpxV/Congo/LNSP-06/2024	EPI_ISL_19345018	Ia
hMpxV/ROC/Cuvette-Centralle/2024/06	hMpxV/Congo/LNSP-017/2024	EPI_ISL_19345029	Ia
hMpxV/ROC/Likouala/2024/07	hMpxV/Congo/LNSP-010/2024	EPI_ISL_19345022	Ia
hMpxV/ROC/Likouala/2024/08	hMpxV/Congo/LNSP-011/2024	EPI_ISL_19345023	Ia
hMpxV/ROC/Likouala/2024/09	hMpxV/Congo/LNSP-08/2024	EPI_ISL_19345020	Ia
hMpxV/ROC/Likouala/2024/010	hMpxV/Congo/LNSP-019/2024	EPI_ISL_19345031	Ia
hMpxV/ROC/Cuvette-Centralle/2024/011	hMpxV/Congo/LNSP-09/2024	EPI_ISL_19345021	Ia
hMpxV/ROC/Cuvette-Centralle/2024/012	hMpxV/Congo/LNSP-020/2024	EPI_ISL_19345032	Ia
hMpxV/ROC/Cuvette-Centralle/2024/013	hMpxV/Congo/LNSP-03/2024	EPI_ISL_19345015	Ia
hMpxV/ROC/Cuvette-Centralle/2024/014	hMpxV/Congo/LNSP-014/2024	EPI_ISL_19345026	Ia
hMpxV/ROC/Cuvette-Centralle/2024/015	hMpxV/Congo/LNSP-04/2024	EPI_ISL_19345016	Ia
hMpxV/ROC/Likouala/2024/16	hMpxV/Congo/LNSP-015/2024	EPI_ISL_19345027	Ia
hMpxV/ROC/Likouala/2024/017	hMpxV/Congo/LNSP-01/2024	EPI_ISL_19345013	Ia
hMpxV/ROC/Likouala/2024/018	hMpxV/Congo/LNSP-012/2024	EPI_ISL_19345024	Ia
hMpxV/ROC/Likouala/2024/019	hMpxV/Congo/LNSP-02/2024	EPI_ISL_19345014	Ia
hMpxV/ROC/Pointe-Noire/2024/020	hMpxV/Congo/LNSP-013/2024	EPI_ISL_19345025	Ia
hMpxV/RoC/Brazzaville/2024/CG000	hMpxV/Congo/BZV-LNSP-CG000/2024	EPI_ISL_19810239	Ib
hMpxV/RoC/Brazzaville/2024/CG001	hMpxV/Congo/BZV-LNSP-CG001/2024	EPI_ISL_19810234	Ib

hMpxV/RoC/Brazzaville/2025/CG018	hMpxV/Congo/BZV-LNSP-CG018/2025	EPI_ISL_19810237	Ib
hMpxV/RoC/Brazzaville/2025/CG020	hMpxV/Congo/BZV-LNSP-CG020/2025	EPI_ISL_19810240	Ib
hMpxV/RoC/Brazzaville/2025/CG021	hMpxV/Congo/BZV-LNSP-CG021/2025	EPI_ISL_19810235	Ib
hMpxV/RoC/Brazzaville/2025/CG022	hMpxV/Congo/BZV-LNSP-CG022/2025	EPI_ISL_19810238	Ib
hMpxV/RoC/Brazzaville/2025/CG031	hMpxV/Congo/BZV-LNSP-CG031/2025	EPI_ISL_19810233	Ib
hMpxV/RoC/Brazzaville/2025/CG032	hMpxV/Congo/BZV-LNSP-CG032/2025	EPI_ISL_19810236	Ib
hMpxV/RoC/Likouala/2023/CG001	hMpxV/Congo/LNSP-CG001/2023	EPI_ISL_20135135	Ia
hMpxV/RoC/Likouala/2023/CG002	hMpxV/Congo/LNSP-CG002/2023	EPI_ISL_20135139	Ia
hMpxV/RoC/Likouala/2023/CG003	hMpxV/Congo/LNSP-CG003/2023	EPI_ISL_20135142	Ia
hMpxV/RoC/Likouala/2023/CG004	hMpxV/Congo/LNSP-CG004/2023	EPI_ISL_20135132	Ia
hMpxV/RoC/Likouala/2023/CG005	hMpxV/Congo/LNSP-CG005/2023	EPI_ISL_20135141	Ia
hMpxV/RoC/Likouala/2023/CG006	hMpxV/Congo/LNSP-CG006/2023	EPI_ISL_20135126	Ia
hMpxV/RoC/Likouala/2023/CG007	hMpxV/Congo/LNSP-CG007/2023	EPI_ISL_20135133	Ia
hMpxV/RoC/Likouala/2023/CG008	hMpxV/Congo/LNSP-CG008/2023	EPI_ISL_20135123	Ia
hMpxV/RoC/Likouala/2023/CG009	hMpxV/Congo/LNSP-CG009/2023	EPI_ISL_20135128	Ia
hMpxV/RoC/Likouala/2023/CG010	hMpxV/Congo/LNSP-CG010/2023	EPI_ISL_20135138	Ia
hMpxV/RoC/Likouala/2023/CG011	hMpxV/Congo/LNSP-CG011/2023	EPI_ISL_20135136	Ia
hMpxV/RoC/Likouala/2023/CG012	hMpxV/Congo/LNSP-CG012/2023	EPI_ISL_20135131	Ia
hMpxV/RoC/Likouala/2023/CG013	hMpxV/Congo/LNSP-CG013/2023	EPI_ISL_20135124	Ia
hMpxV/RoC/Likouala/2023/CG014	hMpxV/Congo/LNSP-CG014/2023	EPI_ISL_20135140	Ia
hMpxV/RoC/Likouala/2023/CG015	hMpxV/Congo/LNSP-CG015/2023	EPI_ISL_20135130	Ia
hMpxV/RoC/Likouala/2023/CG016	hMpxV/Congo/LNSP-CG016/2023	EPI_ISL_20135122	Ia
hMPXV/RoC/Brazzaville/2025/CG056	hMpxV/Congo/LNSP-CG056/2025	EPI_ISL_20135134	Ia
hMPXV/RoC/Brazzaville/2025/CG094	hMpxV/Congo/LNSP-CG094/2025	EPI_ISL_20135129	Ib
hMPXV/RoC/Cuvette-Centrale/2025/CG061	hMpxV/Congo/LNSP-CG061/2025	EPI_ISL_20135127	Ia
hMPXV/RoC/Cuvette-Centrale/2025/CG062	hMpxV/Congo/LNSP-CG062/2025	EPI_ISL_20135125	Ia
hMPXV/RoC/Cuvette-Centrale/2025/CG064	hMpxV/Congo/LNSP-CG064/2025	EPI_ISL_20135137	Ia
hMPXV/RoC/Brazzaville/2025/CG051	hMpxV/Congo/BZV-LNSP-CG051/2025	EPI_ISL_19842849	IIb
